# Burden of stroke in China and the different SDI regions over the world

**DOI:** 10.7189/jogh.13.04169

**Published:** 2023-12-22

**Authors:** Yuqing Mi, Lei Huai, Yanling Yin, Jinbao Yuan, Yuzhuo Liu, Jingwen Huang, Wei Li

**Affiliations:** 1 School of Public Health, Weifang Medical University, Weifang, China; 2 ZIBO TCM- INTEGRATED HOSPITAL, Zibo, China; 3 Qingdao Stomatological Hospital Affiliated to Qingdao University, Qingdao, China; 4 Finance Department, Weifang Medical University, Weifang, China

## Abstract

**Background:**

Stroke is a significant global health issue, ranking as the second leading cause of death and the third leading cause of death and disability combined. This study aimed to examine the changes and differences in stroke burden from 1990 to 2019 in China and various global socio-demographic index (SDI) regions.

**Methods:**

Data were obtained from the Global Burden of Diseases Study 2019, which included the incidence, prevalence, mortality, disability-adjusted life years (DALY), years of life with disability (YLD), and years of life lost (YLL) of stroke. The change trend of stroke burden was assessed based on age-standardised rates per 100 000 person-years and estimated annual percentage changes. The average annual rate of change in stroke burden was analysed using the average annual percentage change from 1990 to 2019. Pearson correlation analysis was used to explore the strength and direction of the correlation between stroke burden and SDI.

**Results:**

Regions with high SDI showed the largest decline in age-standardised incidence, death, DALY, YLD, and YLL rates of stroke from 1990 to 2019. China experienced the largest increase in age-standardised prevalence and YLD rates of stroke from 1990 to 2019. There were significant differences in the average annual percent change in stroke burden among the majority of SDI regions. The burden for stroke at the national level was inversely correlated with SDI, despite some exceptions (Incidence: R = -0.417, *P* < 0.001; prevalence: R = -0.297, *P* < 0.001; mortality: R = -0.510, *P* < 0.001; DALY: R = -0.550, *P* < 0.001; YLD: R = -0.125, *P* = 0.075; YLL: R = -0.569, *P* < 0.001).

**Conclusions:**

There were significant differences in the stroke burden across different regions with varying SDI levels from 1990 to 2019. The age-standardised prevalence rate and attributable disability burden of stroke remain substantial in different SDI regions, making it a major contributor to the overall disease burden. The severe burden of stroke highlights the importance of primary and secondary stroke-prevention strategies. Therefore, future strategies to prevent and reduce the burden of stroke should be formulated and implemented according to the SDI of each country.

Stroke is a common and frequently occurring disease of the nervous system that may lead to various neurological deficits that affect walking, communication, and other activities of daily life [1]. Although considerable progress has been made in the prevention and management of stroke, it remains widespread and burdensome [2]. The 2019 Global Burden of Disease (GBD 2019) reported that stroke is the second leading cause of death and the third leading cause of death and disability worldwide [3]. It is estimated that 33%-42% of patients continue to need daily life assistance six years after stroke, and 36% of patients remain disabled five years after stroke [2]. Stroke costs more than 721 billion US dollars (US$) worldwide [4]. This has created significant social and economic burdens for countries and has become an urgent public health issue.

Stroke can be prevented and rehabilitated, and timely implementation of targeted interventions can improve prognosis. Countries have undertaken various efforts to prevent strokes. For example, China issued the Work Plan for Comprehensive Prevention and Treatment of Stroke [5]. It has been proposed that the construction of stroke prevention and control systems is crucial. Moreover, it clearly puts forward “strengthening the construction of stroke prevention and control system, implementing comprehensive stroke prevention and control strategies and measures, and promoting the transformation from disease treatment to health management” [5]. However, current efforts have been insufficient to achieve the specific goals of sustainable development. The global population is aging, and epidemiological transition continues in all countries [6]. Therefore, it is crucial to evaluate each stroke dimension.

In addition to the GBD 2019 Stroke Collaborators publishing the global stroke burden and its risk factors from 1990 to 2019, numerous studies have reported the stroke burden in various countries, such as China [7], Malaysia [8], Indonesia [9], Cambodia [10], and Myanmar [11]. However, no study has described the changes in stroke burden based on the estimated annual percentage change (EAPC) and average annual percent change (AAPC). The EAPC can provide an estimate of the annual change in the time trend, whereas the AAPC can clarify the total percentage change in the stroke burden from 1990 to 2019 [12]. We retrieved literature on the correlation between social development and the burden of ischemic stroke [13]. Studies have shown that the burden of stroke is related to economic and education level [14]. However, no studies have discussed the relationship between social development and stroke burden. In addition, a Pearson correlation coefficient between stroke burden and Socio-Demographic Index (SDI) were not observed in previous studies.

The GBD 2019 assessed the burden of 369 human diseases and injuries in 204 countries and territories worldwide [15]. In this study, based on the GBD 2019 and SDI data, we aimed to explore the trends and differences in the burden of stroke from 1990 to 2019 in China and various SDI regions worldwide using EAPC and AAPC. Furthermore, we examined the specific relationship between social development, as indicated by the SDI, and stroke burden. The findings could fill the knowledge gap regarding the changes and correlates of stroke burden, providing valuable insights for formulating effective stroke prevention and control strategies and optimising the allocation of medical resources in different SDI regions.

## METHODS

### Data source

Design and standardisation methods for GBD studies have been widely reported, and the quality of the resulting statistics has been recognised internationally [15-18]. The GBD study utilised harmonised and standardised methods to ensure comparability of results, with regional and national representations. GBD is an ongoing global collaborative study that evaluates disease burden according to sex, year, age, cause, and location [19]. In this study, data on the stroke burden from 1990 to 2019 were obtained from the Global Health Data Exchange GBD Results Tool (https://ghdx.healthdata.org/gbd-2019). The obtained data included the age-standardised incidence rate (ASIR), age-standardised prevalence rate (ASPR), age-standardised death rate (ASDR), age-standardised disability-adjusted life years (DALY) rate, age-standardised years lives with disability (YLD) rate, age-standardised years of life lost (YLL) rate globally, in different SDI regions, and in China from 1990 to 2019, and related data in 204 countries (rates per 100 000). The informed consent waiver for the GBD 2019 data was reviewed and approved by the Institutional Review Board of the University of Washington [20].

The SDI reflects the average education level of a country’s population aged 15 and above, lagging income distribution per capita, and fertility rate under 25 years old, which adequately reflects the degree of development of a country; it ranges from 0 (worst) to 1 (best). All regions were divided into five levels according to SDI: high SDI (HSDI), high-middle SDI (HMSDI), middle SDI (MSDI), low-middle SDI (LMSDI), and low SDI (LSDI) [21]. In this study, the SDI was used to confirm the relationship between a country’s degree of socioeconomic development and stroke burden. SDI data from 1990 to 2019 were obtained from the Global Health Data Exchange Results Tool (http://ghdx.healthdata.org/record/ihme-data/gbd-2019-socio-demographic-index-sdi-1950-2019). Ethical approval was waived because SDI data were publicly available.

### Statistical analysis

Because the data were related to age structures and populations in different periods, this study adopted an age-standardised estimation. The age-standardised rate (ASR) per 100 000 individuals was calculated as follows [22]:



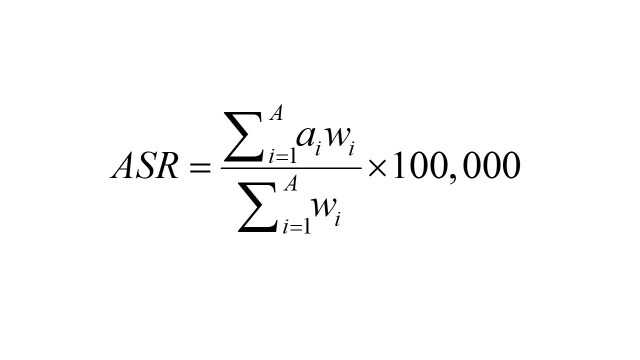



*A*: the number of age groups; *i*: the *i^th^* age group;

*a_i_*: the age-specific rate of the *i^th^* age group;

*w_i_*: the population numbers in the corresponding *i_th_* age group among the GBD standard population.

In this study, the EAPC was used to quantify ASR changes. To estimate the EAPC, the natural logarithm of the ASR should be calculated to regress linearly with time [23,24], which is expressed as follows: *y = α + βx + ε*

*y*: the natural logarithm of ASR; *x*: the corresponding calendar year.

Next, the linear regression model was used to estimate the EAPC and its 95% confidence interval (CI). If EAPC>0 and 95% CI>0, which are believed to be an increasing trend. If EAPC<0 and 95% CI<0, it is considered to be a declining trend. If the 95% CI crosses 0, the changes are considered to have no reference value [25]. The formula is as follows: EAPC = 100 × [exp (β) - 1].

The average annual rate of change in stroke burden was analysed using the AAPC from 1990 to 2019. The following formula was used [26]:


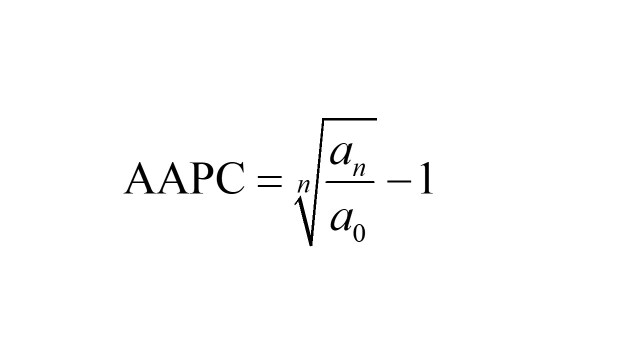
.

Joinpoint models (JM) were established, with the independent variables being time, and the dependent variables being ASIR, ASPR, ASDR, age-standardised DALY/YLD/YLL rate. Whether the AAPC between different SDI regions was statistically significant was tested using pairwise comparisons.

Pearson correlation analysis was used to explore the strength and direction of the correlation between the ASR and SDI. Multiple levels of statistical significance were established, with *P*-values indicated as “*P* <0.001” for highly significant, “*P* <0.01” for significant, and “*P* <0.05” for marginally significant, and “P <0.1” for barely significant.

## RESULTS

### Analysis on the burden and trends of stroke

**Figure 1:**
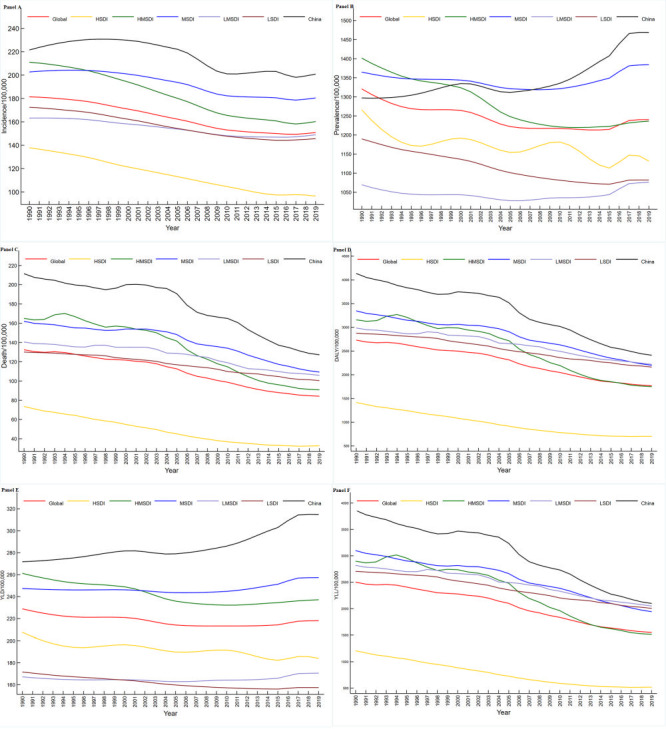
Trends of burden for stroke in China and the different SDI regions over the world from 1990 to 2019. **Panel A.** Trends of ASIR for stroke in China and the different SDI regions over the world from 1990 to 2019. **Panel B.** Trends of ASPR for stroke in China and the different SDI regions over the world from 1990 to 2019. **Panel C.** Trends of ASDR for stroke in China and the different SDI regions over the world from 1990 to 2019. **Panel D.** Trends of the age-standardised DALY rate for stroke in China and the different SDI regions over the world from 1990 to 2019. **Panel E.** Trends of the age-standardised YLD rate for stroke in China and the different SDI regions over the world from 1990 to 2019. **Panel F.** Trends of the age-standardised YLL rate for stroke in China and the different SDI regions over the world from 1990 to 2019.

**Table 1** and **Tabel 2** showed the ASIR, ASPR, ASDR, and age-standardised DALY/YLD/YLL rates with their EAPC from 1990 to 2019 for different sexes in China and different SDI regions in the world. In the MSDI and LMSDI regions and China, the ASPR for stroke showed an increasing trend (EAPC>0), and the additional disease burden indicators in all areas showed a declining trend (EAPC<0). Trends in the stroke burden from 1990 to 2019 are shown **in** **Figure 1**.

**Table 1:** Trends of ASIR, ASPR, ASDR of stroke in China and the different SDI regions over the world from 1990 to 2019, with EAPC from 1990 to 2019

Year	ASIR (1/100 000) (95% CI)			ASPR (1/100 000) (95% CI)			ASDR (1/100 000) (95% CI)		
	Male	Female	Both	Male	Female	Both	Male	Female	Both
Globe									
**1990**	**178.54 (162.74-196.76)**	**182.05 (165.70-201.28)**	**181.40 (165.21-199.76)**	**1199.56 (1098.92-1315.82)**	**1418.62 (1305.83-1542.25)**	**1320.79 (1212.44-1440.50)**	**143.14 (134.11-153.21)**	**122.92 (111.67-133.58)**	**132.44 (123.08-141.70)**
**2019**	**151.10 (136.90-167.54)**	**149.75 (135.58-166.56)**	**150.77 (136.52-167.46)**	**1150.19 (1052.72-1259.3)**	**1316.73 (1210.72-1433.71)**	**1240.26 (1139.71-1352.99)**	**96.36 (87.63-104.21)**	**73.50 (65.21-80.66)**	**84.19 (76.76-90.15)**
**EAPC (%)**	**-0.75 (-0.81,-0.69)**	**-0.83 (-0.89,-0.78)**	**-0.80 (-0.86,-0.75)**	**-0.13 (-0.18,-0.09)**	**-0.29 (-0.35,-0.22)**	**-0.23 (-0.28,-0.17)**	**-1.49 (-1.61,-1.37)**	**-2.00 (-2.13,-1.86)**	**-1.74 (-1.86,-1.61)**
HSDI regions									
**1990**	**134.98 (122.40-149.78)**	**138.32 (125.32-153.97)**	**137.84 (124.87-152.99)**	**1184.94 (1082.07-1304.66)**	**1329.65 (1223.59-1444.01)**	**1266.03 (1163.59-1383.61)**	**82.18 (77.79-84.59)**	**66.98 (60.76-70.26)**	**73.53 (67.90-76.41)**
**2019**	**91.95 (83.71-101.11)**	**99.64 (90.11-110.09)**	**96.47 (87.65-106.10)**	**1057.17 (967.47-1161.01)**	**1199.12 (1105.34-1298.30)**	**1131.82 (1041.34-1229.27)**	**36.56 (33.19-38.71)**	**29.00 (24.50-31.64)**	**32.63 (28.39-35.06)**
**EAPC (%)**	**-1.49 (-1.55,-1.43)**	**-1.23 (-1.28,-1.19)**	**-1.36 (-1.41, -1.31)**	**-0.17 (-0.26,-0.08)**	**-0.29 (-0.35,-0.23)**	**-0.25 (-0.32,-0.19)**	**-3.15 (-3.30,-2.99)**	**-3.23 (-3.38,-3.07)**	**-3.14 (-3.29,-2.99)**
HMSDI regions									
**1990**	**209.81 (190.32-231.95)**	**209.14 (188.26-233.86)**	**211.03 (190.31-234.15)**	**1276.77 (1164.09-1408.79)**	**1494.81 (1366.23-1635.29)**	**1401.51 (1283.38-1533.07)**	**180.05 (168.38-92.31)**	**152.68 (140.75-163.2)**	**164.98 (153.49-174.73)**
**2019**	**164.59 (147.80-185.32)**	**155.38 (138.69-175.30)**	**160.20 (143.77-180.69)**	**1145.90 (1041.52-1264.94)**	**1308.98 (1188.35-1432.73)**	**1236.33 (1126.68-1358.28)**	**107.14 (95.66-117.84)**	**77.74 (68.32-86.18)**	**90.86 (81.83-98.27)**
**EAPC (%)**	**-1.07 (-1.16,-0.99)**	**-1.22 (-1.29,-1.16)**	**-1.16 (-1.23,-1.09)**	**-0.46 (-0.53,-0.40)**	**-0.52 (-0.60,-0.44)**	**-0.50 (-0.57,-0.43)**	**-2.06 (-2.28,-1.83)**	**-2.79 (-3.05,-2.53)**	**-2.44 (-2.68,-2.20)**
MSDI regions									
**1990**	**200.45 (181.20-223.25)**	**203.55 (184.22-226.55)**	**202.69 (183.22-224.87)**	**1219.54 (1114.23-1345.01)**	**1492.81 (1366.79-1636.86)**	**1364.92 (1249.00-1498.62)**	**170.84 (154.50-190.81**	**153.32 (136.14-174.77)**	**162.02 (147.34-181.15)**
**2019**	**184.23 (166.16-205.81)**	**176.56 (157.46-199.09)**	**180.42 (161.94-202.86)**	**1275.19 (1162.26-1406.46)**	**1477.14 (1350.46-1618.28)**	**1384.62 (1266.53-1518.05)**	**127.66 (112.49-142.57)**	**92.92 (80.58-105.25)**	**109.24 (98.32-119.45)**
**EAPC (%)**	**-0.45 (-0.52,-0.38)**	**-0.67 (-0.73,-0.61)**	**-0.57 (-0.63,-0.51)**	**0.11 (0.05, 0.18)**	**-0.08 (-0.13,-0.02)**	**0.01 (-0.05,0.07)**	**-0.96 (-1.11,-0.81)**	**-1.76 (-1.94,-1.58)**	**-1.35 (-1.52,-1.19)**
LMSDI regions									
**1990**	**160.99 (147.33-178.06)**	**164.91 (151.41-180.18)**	**163.22 (149.78-179.08)**	**984.59 (900.35-1077.08)**	**1152.66 (1062.40-1252.34)**	**1069.59 (982.67-1164.66)**	**146.82 (133.16-160.94)**	**133.67 (114.74-153.61)**	**140.49 (126.77-158.01)**
**2019**	**148.62 (135.87-163.56)**	**149.05 (135.95-164.41)**	**148.92 (136.20-164.08)**	**1011.44 (924.54-1110.36)**	**1135.74 (1044.11-1231.88)**	**1076.19 (986.72-1169.51)**	**115.96 (104.94-127.64)**	**96.78 (85.35-108.11)**	**105.89 (96.45-114.62)**
**EAPC (%)**	**-0.39 (-0.43,-0.35)**	**-0.50 (-0.56,-0.44)**	**-0.45 (-0.49,-0.41)**	**0.10(0.06, 0.14)**	**-0.09 (-0.15,-0.02)**	**0.01 (-0.05,0.06)**	**-0.87 (-0.99,-0.74)**	**-1.20 (-1.31,-1.09)**	**-1.04 (-1.16,-0.93)**
LSDI regions									
**1990**	**168.53 (155.46-184.39)**	**176.03 (162.99-191.55)**	**172.45 (159.37-188.12)**	**1084.73 (997.46-1176.10)**	**1293.72 (1197.63-1396.03)**	**1189.79 (1100.37-1285.98)**	**135.95 (118.44-154.25)**	**123.72 (93.85-151.78)**	**130.03 (109.45-152.02)**
**2019**	**140.82 (129.63-154.18)**	**150.05 (138.55-162.95)**	**145.63 (134.46-158.83)**	**989.06 (910.42-1072.38)**	**1171.12 (1080.60-1260.43)**	**1082.06 (1000.51-1166.31)**	**102.59 (88.84-117.66)**	**98.21 (84.29-112.30)**	**100.42 (87.65-114.15)**
**EAPC (%)**	**-0.75 (-0.79,-0.71)**	**-0.71 (-0.77,-0.64)**	**-0.73 (-0.78,-0.67)**	**-0.35 (-0.39,-0.31)**	**-0.40 (-0.45,-0.36)**	**-0.37 (-0.41,-0.33)**	**-1.04 (-1.10,-0.97)**	**-0.87 (-0.92,-0.83)**	**-0.97 (-1.02,-0.91)**
China									
**1990**	**227.92 (202.83-257.34)**	**216.45 (192.14-245.19)**	**221.51 (196.81-249.61)**	**1139.75 (1030.82-1269.03)**	**1421.08 (1279.80-1579.43)**	**1297.10 (1173.49-1439.18)**	**246.30 (212.62-286.86)**	**188.28 (161.70-224.20)**	**211.44 (187.68-243.80)**
**2019**	**209.75 (185.84-239.39)**	**194.53 (169.65-225.19)**	**200.84 (176.95-230.84)**	**1325.35 (1181.03-1486.04)**	**1583.53 (1403.67-1771.38)**	**1468.87 (1309.21-1640.07)**	**170.32 (141.87-200.18)**	**97.44 (78.87-117.01)**	**127.25 (110.21-144.89)**
**EAPC (%)**	**-0.57 (-0.73,-0.41)**	**-0.58 (-0.66, -0.50)**	**-0.59 (-0.71,-0.48)**	**0.44 (0.34,0.54)**	**0.33 (0.25,0.42)**	**0.37 (0.29,0.46)**	**-1.17 (-1.38,-0.97)**	**-2.36 (-2.62,-2.10)**	**-1.77 (-2.00,-1.54)**


CI – confidence interval, EAPC – estimated annual percentage change, ASIR – age-standardised incidence rate, ASPR – age-standardised prevalence rate, ASDR – age-standardised death rate, SDI – socio-demographic index, HSDI – high SDI, HMSDI – high-middle SDI, MSDI – middle SDI, LMSDI – low-middle SDI, LSDI – low SDI

**Table 2:** Trends of DALY, YLD, YLL of stroke in China and the different SDI regions over the world from 1990 to 2019, with EAPC from 1990 to 2019

Year	DALY (1/100 000) (95% CI)			YLD (1/100 000) (95% CI)			YLL (1/100 000) (95% CI)		
	**Male**	**Female**	**Both**	**Male**	**Female**	**Both**	**Male**	**Female**	**Both**
**Globe**									
1990	2976.71 (2783.09-3183.15)	2502.24 (2317.01-2687.22)	2729.87 (2579.92-2901.17)	191.94 (138.97-245.32)	258.87 (186.84-327.80)	228.82 (165.32-291.01)	2784.78 (2599.05-2972.52)	2243.37 (2069.25-2421.40)	2501.05 (2364.43-2660.06)
2019	2024.28 (1852.42-2195.62)	1531.27 (1397.07-1667.60)	1768.05 (1640.65-1889.39)	187.96 (135.87-239.86)	243.90 (174.98-308.49)	218.07 (156.65-276.98)	1836.32 (1670.42-1995.94)	1287.37 (1164.08-1407.76)	1549.99 (1434.51-1660.71)
EAPC (%)	-1.43 (-1.54,-1.33)	-1.90 (-2.01,-1.79)	-1.65 (-1.75,-1.54)	-0.07 (-0.12,-0.03)	-0.24 (-0.30,-0.17)	-0.18 (-0.24,-0.13)	-1.54 (-1.66,-1.43)	-2.14 (-2.27,-2.01)	-1.81 (-1.93,-1.69)
**HSDI regions**									
1990	1588.59 (1519.14-1648.37)	1274.88 (1188.82-1348.58)	1415.78 (1335.19-1483.76)	181.98 (132.23-232.65)	226.88 (163.51-286.68)	207.50 (150.39-263.64)	1406.61 (1355.52-1438.43)	1048.00 (980.93-1083.24)	1208.28 (1146.78-1240.40)
2019	775.13 (717.92-831.32)	634.85 (566.90-697.43)	703.13 (640.03-761.95)	161.35 (117.06-204.97)	204.19 (146.53-257.47)	184.09 (132.85-232.1)	613.78 (574.91-649.99)	430.66 (385.99-462.97)	519.04 (478.40-551.17)
EAPC (%)	-2.72 (-2.85,-2.60)	-2.66 (-2.80,-2.52)	-2.66 (-2.79,-2.53)	-0.23 (-0.30,-0.16)	-0.32 (-0.37,-0.28)	-0.32 (-0.37,-0.27)	-3.19 (-3.34,-3.04)	-3.43 (-3.60,-3.26)	-3.25 (-3.41,-3.10)
**HMSDI regions**									
1990	3529.27 (3287.68-3772.26)	2844.53 (2651.48-3027.91)	3158.39 (2985.92-3325.54)	218.8 (158.93-279.23)	293.26 (210.81-374.43)	261.17 (187.59-334.16)	3310.47 (3076.26-3546.74)	2551.27 (2371.17-2720.51)	2897.22 (2741.21-3049.85)
2019	2079.62 (1867.15-2304.51)	1462.13 (1317.49-1605.46)	1750.21 (1606.12-1882.09)	204.47 (146.54-263.27)	263.92 (188.60-335.75)	237.13 (169.72-302.55)	1875.16 (1670.48-2086.28)	1198.21 (1068.85-1330.44)	1513.08 (1380.79-1630.84)
EAPC(%)	-2.12 (-2.35,-1.89)	-2.75 (-2.99,-2.50)	-2.41 (-2.65,-2.17)	-0.33 (-0.39,-0.26)	-0.42 (-0.50,-0.34)	-0.40 (-0.47,-0.32)	-2.27 (-2.52,-2.01)	-3.10 (-3.39,-2.81)	-2.64 (-2.91,-2.37)
**MSDI regions**									
1990	3554.25 (3218.42-3918.00)	3139.23 (2841.64-3483.18)	3346.69 (3100.07-3654.44)	203.79 (146.51-261.18)	286.17 (206.22-366.86)	247.42 (178.73-317.23)	3350.46 (3016.18-3714.15)	2853.07 (2556.22-3202.03)	3099.27 (2858.55-3405.75)
2019	2578.24 (2304.27-2873.26)	1848.14 (1652.39-2064.34)	2199.78 (2017.08-2393.22)	219.26 (157.37-280.71)	290.16 (205.96-370.99)	257.25 (184.30-328.94)	2358.98 (2079.92-2643.55)	1557.98 (1370.52-1764.75)	1942.53 (1765.06-2123.50)
EAPC (%)	-1.05 (-1.16,-0.93)	-1.88 (-2.00,-1.76)	-1.44 (-1.55,-1.32)	0.21 (0.15,0.27)	0.02 (-0.03,0.07)	0.10 (0.05,0.16)	-1.14 (-1.26,-1.01)	-2.13 (-2.27,-1.99)	-1.59 (-1.72,-1.46)
**LMSDI regions**									
1990	3154.12 (2875.66-3425.69)	2807.8 (2493.67-3150.73)	2985.86 (2744.66-3268.01)	142.37 (103.04-181.56)	191.75 (139.40-242.52)	167.23 (121.73-211.86)	3011.75 (2736.54-3281.63)	2616.05 (2301.59-2950.83)	2818.64 (2581.94-3099.11)
2019	2443.84 (2208.55-2688.16)	2010.72 (1795.22-2240.43)	2220.33 (2036.51-2414.45)	148.59 (107.40-189.35)	190.80 (137.71-241.04)	170.60 (123.56-217.03)	2295.25 (2060.89-2539.52)	1819.93 (1610.62-2046.07)	2049.73 (1873.76-2229.74)
EAPC(%)	-0.90 (-1.00,-0.80)	-1.23 (-1.31,-1.15)	-1.07 (-1.15,-0.99)	0.15 (0.11, 0.19)	-0.05 (-0.11,0.01)	0.05 (0.00, 0.10)	-0.96 (-1.06,-0.85)	-1.33 (-1.42,-1.24)	-1.15 (-1.24,-1.05)
**LSDI regions**									
1990	3017.67 (2667.82-3382.51)	2726.05 (2183.56-3256.78)	2877.01 (2510.42-3267.86)	144.85 (106.07-181.81)	198.31 (144.30-247.98)	171.58 (125.54-214.63)	2872.82 (2527.28-3241.91)	2527.74 (1992.26-3049.48)	2705.43 (2347.41-3083.52)
2019	2225.29 (1934.12-2550.34)	2097.54 (1838.03-2367.28)	2162.03 (1912.56-2441.68)	133.09 (96.94-167.92)	180.69 (132.48-227.10)	157.42 (115.09-197.14)	2092.19 (1804.52-2415.20)	1916.84 (1662.16-2186.36)	2004.60 (1757.71-2274.59)
EAPC (%)	-1.13 (-1.19,-1.08)	-1.00 (-1.04,-0.96)	-1.08 (-1.12,-1.03)	-0.31 (-0.35,-0.27)	-0.37 (-0.41,-0.33)	-0.33 (-0.37,-0.30)	-1.18 (-1.24,-1.12)	-1.05 (-1.10,-1.01)	-1.13 (-1.18,-1.08)
**China**									
1990	4656.60 (3971.22-5403.45)	3707.97 (3178.76-4330.48)	4134.28 (3697.14-4674.62)	221.97 (158.79-287.35)	313.17 (222.76-403.90)	271.73 (194.37-349.40)	4434.63 (3744.50-5186.58)	3394.80 (2884.17-4016.16)	3862.55 (3426.57-4387.25)
2019	3052.05 (2535.20-3643.00)	1876.80 (1566.50-2204.53)	2412.52 (2102.92-2742.48)	264.23 (187.75-342.72)	357.64 (249.21-461.40)	314.85 (220.62-407.81)	2787.83 (2282.60-3350.75)	1519.16 (1222.87-1843.60)	2097.67 (1801.17-2421.81)
EAPC(%)	-1.41 (-1.58,-1.24)	-2.47 (-2.67,-2.28)	-1.90 (-2.07,-1.72)	0.53 (0.44,0.63)	0.43 (0.36,0.51)	0.47 (0.38,0.55)	-1.54 (-1.72,-1.36)	-2.89 (-3.14,-2.65)	-2.13 (-2.34,-1.93)

CI – confidence interval, EAPC – estimated annual percentage change, DALY – disability-adjusted life yeas, YLD – lives with disability, YLL – years of life lost, SDI – socio-demographic index, HSDI – high SDI, HMSDI – high-middle SDI, MSDI – middle SDI, LMSDI – low-middle SDI, LSDI – low SDI.

#### Incidence

**Figure 2:**
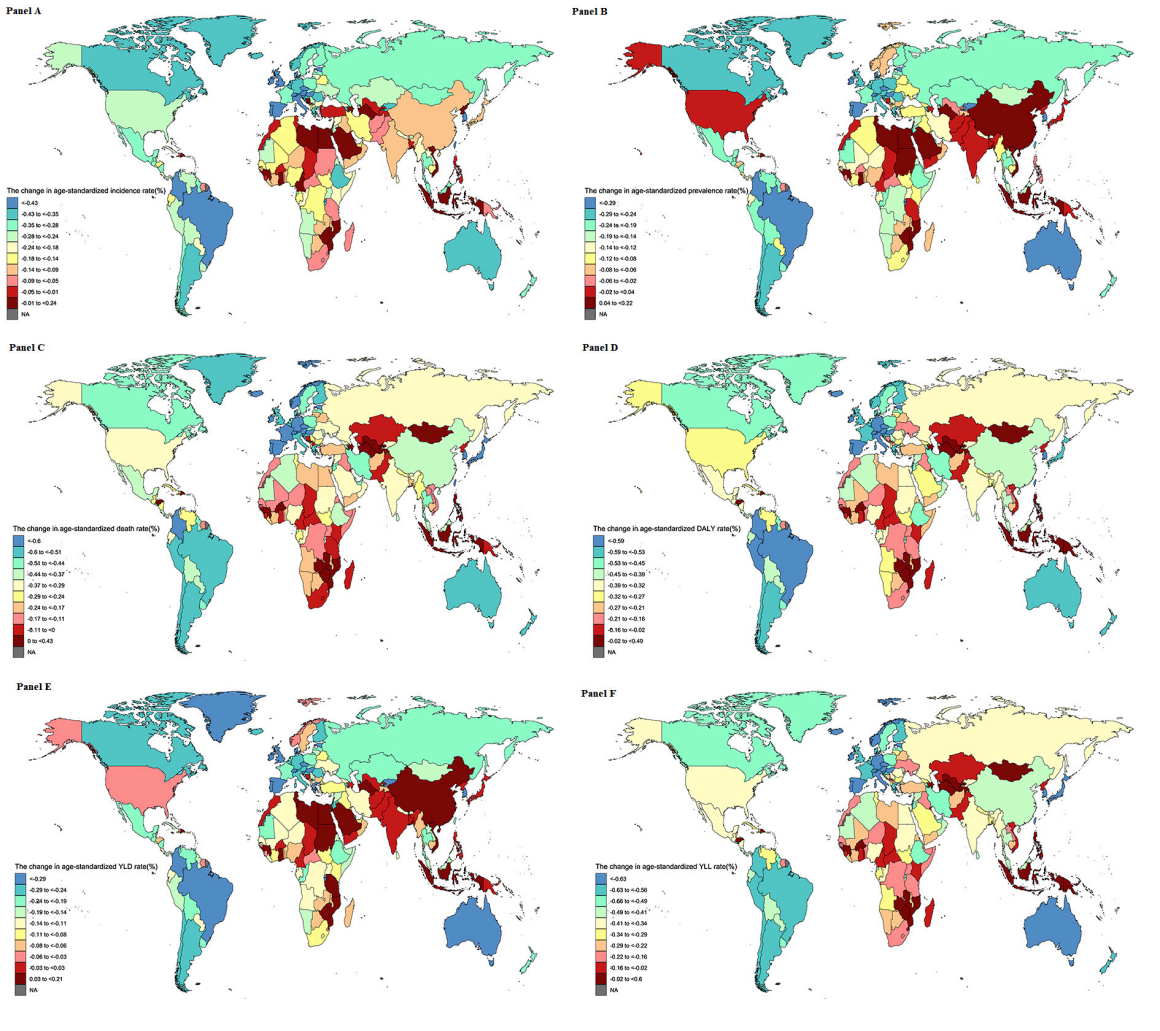
Changes in burden of stroke over the world from 1990 to 2019. **Panel A.** Changes in ASIR of stroke over the world from 1990 to 2019. **Panel B.** Changes in ASPR of stroke over the world from 1990 to 2019. **Panel C.** Changes in ASDR of stroke over the world from 1990 to 2019. **Panel D.** Changes in the age-standardised DALY rate of stroke over the world from 1990 to 2019. **Panel E.** Changes in the age-standardised YLD rate of stroke over the world from 1990 to 2019. **Panel F.** Changes in the age-standardised YLL rate of stroke over the world from 1990 to 2019.

The final selected JM for the ASIR of stroke showed four joinpoints in the global and HMSDI regions, five joinpoints in China, and three joinpoints in other regions (**Table 3**). The HSDI regions changed the most in the ASIR of stroke between 1990 and 2019 (AAPC = -1.217, 95% CI = -1.258 to -1.176, *P* < 0.001), with China experiencing a greater increase from 1990 to 1997 than the other regions (**Table 3** and **Figure 1**, panel A). Through pairwise comparison analysis, the difference in the AAPC of stroke ASIR between China and the MSDI/LMSDI regions from 1990 to 2019 was not statistically significant (China and MSDI: AAPC = 0.052, 95% CI = -0.086 to 0.190, *P* = 0.458; China and LMSDI: AAPC = -0.035, 95% CI = -0.166 to 0.097, *P* = 0.606), while the AAPC differences between the other two regions were significant (**Table 4** and **Figure 2**, panel A).

**Table 3:** Changes of ASIR, ASPR, ASDR of stroke in China and the different SDI regions over the world from 1990 to 2019, with AAPC from 1990 to 2019

	ASIR			ASPR			ASDR		
	**Js**	**AAPC (95% CI)**	***P*-value**	**Js**	**AAPC (95% CI)**	***P*-value**	**Js**	**AAPC (95% CI)**	***P*-value**
**Global**									
Male	4	-0.584 (-0.620,-0.548)	<0.001	4	-0.121 (-0.227,-0.013)	0.027	2	-1.380 (-1.481,-1.279)	<0.001
Female	5	-0.676 (-0.705,-0.646)	<0.001	4	-0.244 (-0.278,-0.210)	<0.001	5	-1.757 (-1.944,-1.569)	<0.001
Both	4	-0.642 (-0.668,-0.616)	<0.001	4	-0.199 (-0.250,-0.149)	<0.001	3	-1.579 (-1.710,-1.447)	<0.001
**HSDI regions**									
Male	5	-1.311 (-1.366,-1.256)	<0.001	5	-0.299 (-0.589,-0.008)	0.044	4	-2.753 (-2.898,-2.607)	<0.001
Female	4	-1.106 (-1.204,-1.009)	<0.001	5	-0.328 (-0.398,-0.258)	<0.001	5	-2.827 (-3.077,-2.576)	<0.001
Both	3	-1.217 (-1.258,-1.176)	<0.001	5	-0.329 (-0.508,-0.150)	<0.001	4	-2.770 (-2.918,-2.622)	<0.001
**HMSDI regions**									
Male	5	-0.842 (-0.914,-0.771)	<0.001	5	-0.362 (-0.397,-0.327)	<0.001	4	-1.770 (-2.128,-1.41)	<0.001
Female	5	-1.024 (-1.059,-0.989)	<0.001	5	-0.455 (-0.478,-0.431)	<0.001	4	-2.287 (-2.603,-1.969)	<0.001
Both	4	-0.956 (-1.009,-0.904)	<0.001	5	-0.427 (-0.449,-0.405)	<0.001	4	-2.015 (-2.336,-1.692)	<0.001
**MSDI regions**									
Male	5	-0.298 (-0.349,-0.247)	<0.001	5	0.158 (0.122,0.193)	<0.001	4	-1.026 (-1.179,-0.873)	<0.001
Female	5	-0.491 (-0.522,-0.460)	<0.001	4	-0.025 (-0.068,0.018)	0.252	5	-1.721 (-1.886,-1.555)	<0.001
Both	5	-0.407 (-0.457,-0.356)	<0.001	5	0.053 (0.003,0.102)	0.036	5	-1.349 (-1.507,-1.192)	<0.001
**LMSDI regions**									
Male	5	-0.280 (-0.353,-0.207)	<0.001	5	0.098 (0.064,0.131)	<0.001	3	-0.799 (-1.030,-0.567)	<0.001
Female	5	-0.348 (-0.372,-0.324)	<0.001	5	-0.045 (-0.076,-0.014)	0.005	3	-1.105 (-1.227,-0.982)	<0.001
Both	5	-0.320 (-0.346,-0.294)	<0.001	5	0.027 (-0.012,0.065)	0.172	3	-0.943 (-1.073,-0.813)	<0.001
**LSDI regions**									
Male	5	-0.624 (-0.647,-0.600)	<0.001	4	-0.313 (-0.336,-0.29)	<0.001	2	-0.979 (-1.038,-0.919)	<0.001
Female	5	-0.551 (-0.578,-0.524)	<0.001	4	-0.333 (-0.365,-0.300)	<0.001	5	-0.805 (-0.943,-0.668)	<0.001
Both	5	-0.591 (-0.61,-0.572)	<0.001	4	-0.319 (-0.346,-0.291)	<0.001	1	-0.905 (-0.950,-0.861)	<0.001
**China**									
Male	5	-0.302 (-0.402,-0.202)	<0.001	5	0.531 (0.477,0.586)	<0.001	4	-1.295 (-1.523,-1.066)	<0.001
Female	5	-0.378 (-0.477,-0.279)	<0.001	5	0.378 (0.349,0.406)	<0.001	5	-2.258 (-2.603,-1.911)	<0.001
Both	5	-0.355 (-0.483,-0.226)	<0.001	5	0.441 (0.369,0.512)	<0.001	5	-1.732 (-2.026,-1.437)	<0.001

Js – Joinpoints, CI – confidence interval, AAPC – average annual percent change, ASIR – age-standardised incidence rate, ASPR – age-standardised prevalence rate, ASDR – age-standardised death rate, SDI – socio-demographic index, HSDI – high SDI, HMSDI – high-middle SDI, MSDI – middle SDI, LMSDI – low-middle SDI, LSDI – low SDI.

**Table 4:** AAPC differences of ASIR, ASPR, ASDR of stroke in China and the different SDI regions over the world from 1990 to 2019

		ASIR		ASPR		ASDR	
**Location**		**Differences (95% CI)**	***P*-value**	**Differences (95% CI)**	***P*-value**	**Differences (95% CI)**	***P*-value**
China	Global	0.287 (0.156-0.418)	<0.001	0.640 (0.552-0.728)	<0.001	-0.154 (-0.476,0.169)	0.351
China	HSDI	0.862 (0.728-0.997)	<0.001	0.770 (0.577-0.963)	<0.001	1.038 (0.708-1.368)	<0.001
China	HMSDI	0.602 (0.463-0.741)	<0.001	0.867 (0.793-0.942)	<0.001	0.283 (-0.154,0.719)	0.204
China	MSDI	0.052 (-0.086,0.190)	0.458	0.388 (0.301-0.475)	<0.001	-0.383 (-0.717,-0.048)	0.025
China	LMSDI	-0.035 (-0.166,0.097)	0.606	0.414 (0.333-0.495)	<0.001	-0.789 (-1.111,-0.467)	<0.001
China	LSDI	0.236 (0.106-0.366)	<0.001	0.759 (0.683-0.836)	<0.001	-0.827 (-1.125,-0.529)	<0.001
Global	HSDI	0.575 (0.527-0.624)	<0.001	0.130 (-0.057,0.316)	0.173	1.191 (0.993-1.390)	<0.001
Global	HMSDI	0.315 (0.256-0.373)	<0.001	0.227 (0.172-0.283)	<0.001	0.436 (0.089-0.784)	0.014
Global	MSDI	-0.235 (-0.292,-0.178)	<0.001	-0.252 (-0.323,-0.182)	<0.001	-0.229 (-0.435,-0.024)	0.029
Global	LMSDI	-0.322 (-0.359,-0.285)	<0.001	-0.226 (-0.290,-0.163)	<0.001	-0.635 (-0.820,-0.450)	<0.001
Global	LSDI	-0.051 (-0.083,-0.018)	0.002	0.119 (0.062-0.177)	<0.001	-0.673 (-0.812,-0.534)	<0.001
HSDI	HMSDI	0.260 (0.194-0.327)	<0.001	-0.098 (-0.278,0.083)	0.290	0.755 (0.401-1.109)	<0.001
HSDI	MSDI	-0.810 (-0.875,-0.745)	<0.001	-0.382 (-0.568,-0.196)	<0.001	-1.420 (-1.637,-1.204)	<0.001
HSDI	LMSDI	-0.897 (-0.945,-0.849)	<0.001	-0.356 (-0.539,-0.173)	<0.001	-1.827 (-2.024,-1.630)	<0.001
HSDI	LSDI	-0.626 (-0.671,-0.581)	<0.001	-0.011 (-0.192,0.171)	0.909	-1.865 (-2.019,-1.710)	<0.001
HMSDI	MSDI	-0.550 (-0.622,-0.477)	<0.001	-0.479 (-0.533,-0.426)	<0.001	-0.665 (-1.024,-0.307)	<0.001
HMSDI	LMSDI	-0.637 (-0.695,-0.578)	<0.001	-0.454 (-0.498,-0.409)	<0.001	-1.071 (-1.408,-0.724)	<0.001
HMSDI	LSDI	-0.366 (-0.421,-0.310)	<0.001	-0.108 (-0.143,-0.073)	<0.001	-1.110 (-1.434,-0.785)	<0.001
MSDI	LMSDI	0.087 (0.030-0.144)	0.003	-0.026 (-0.088,0.037)	0.418	0.406 (0.202-0.610)	<0.001
MSDI	LSDI	-0.184 (-0.238,-0.130)	<0.001	-0.371 (-0.427,-0.315)	<0.001	0.444 (0.280-0.608)	<0.001
LMSDI	LSDI	0.271 (0.238-0.303)	<0.001	0.345 (0.298-0.393)	<0.001	-0.038 (-0.175,0.099)	0.586

CI – confidence interval, AAPC – average annual percent change, ASIR – an age-standardised incidence rate, ASPR – an age-standardised prevalence rate, ASDR – an age-standardised death rate, SDI – socio-demographic index, HSDI – high SDI, HMSDI – high-middle SDI, MSDI – middle SDI, LMSDI – low-middle SDI, LSDI – low SDI

#### Prevalence

The final selected JM for the ASPR of stroke showed four joinpoints in the global and LSDI regions and five joinpoints in other regions (**Table 3**). The biggest increase in ASPR of stroke between 1990 and 2019 was in China (AAPC = 0.441, 95% CI = 0.369 to 0.512, *P* < 0.001), with a higher decline in HMSDI regions (AAPC = -0.427, 95% CI = -0.449 to -0.405, *P* < 0.001; **Table 3** and **Figure 1**, panel B). Through pairwise comparison analysis, the AAPC difference in the ASPR of stroke from 1990 to 2019 was not statistically significant between the HSDI and global/ HMSDI/ LSDI MSDI and LMSDI regions (**Table 4** and **Figure 2**, panel B).

#### Death

The final selected JM for the ASDR of stroke revealed three joinpoints in the global and LMSDI regions, four joinpoints in the HSDI and HMSDI regions and five joinpoints in the China and MSDI regions (**Table 3**). The HSDI regions experienced the largest decline in the ASDR for stroke from 1990 to 2019 (AAPC = -2.770, 95% CI = -2.918 to -2.622, *P* < 0.001), with a higher rate of decline from 2004 to 2007 in China than in other regions (**Table 3** and **Figure 1**, panel C). The results of the pairwise comparison analysis showed that the difference in AAPC for ASDR of stroke from 1990 to 2019 was not statistically significant between China and the global/HMSDI regions, LMSDI and LSDI regions (**Table 4** and **Figure 2**, panel C).

#### DALY

The age-standardised DALY rate of stroke exhibited five joinpoints in the global, Chin, and MSDI regions; three joinpoints in the HSDI regions; and four joinpoints in the HMSDI regions. Two joinpoints were observed in the LMSDI and LSDI regions (**Table 5**). The largest decline in the DALY rate of stroke between 1990 and 2019 was in the HSDI regions (AAPC = -2.384, 95% CI = -2.469 to -2.299, *P* < 0.001) and the HMSDI regions showed a greater decline from 2003 to 2013 than the other regions (**Table 5** and **Figure 1**, panel D). The results of the pairwise comparison analysis revealed that the difference in AAPC for the age-standardised DALY rate of stroke from 1990 to 2019 was not statistically significant between China and the HMSDI regions, the globe and MSDI regions, and the LMSDI and LSDI regions, while the AAPC differences between any other two regions were found to be statistically significant (**Table 6** and **Figure 2**, panel D).

**Table 5:** Changes of DALY, YLD, YLL of stroke in China and the different SDI regions over the world from 1990 to 2019, with AAPC from 1990 to 2019

	DALY			YLD			YLL		
	**Js**	**AAPC (95% CI)**	***P*-value**	**Js**	**AAPC (95% CI)**	***P*-value**	**Js**	**AAPC (95% CI)**	***P*-value**
**Global**									
Male	2	-1.341 (-1.440,-1.243)	<0.001	5	-0.066 (-0.113,-0.018)	0.007	2	-1.452 (-1.559,-1.345)	<0.001
Female	5	-1.669 (-1.813,-1.525)	<0.001	5	-0.197 (-0.229,-0.166)	<0.001	5	-1.888 (-2.054,-1.721)	<0.001
Both	5	-1.479 (-1.625,-1.332)	<0.001	5	-0.161 (-0.187,-0.135)	<0.001	5	-1.633 (-1.773,-1.493)	<0.001
**HSDI regions**									
Male	5	-2.448 (-2.568,-2.328)	<0.001	5	-0.358 (-0.618,-0.097)	0.007	4	-2.822 (-2.939,-2.705)	<0.001
Female	3	-2.377 (-2.472,-2.281)	<0.001	5	-0.348 (-0.396,-0.299)	<0.001	4	-3.040 (-3.189,-2.89)	<0.001
Both	3	-2.384 (-2.469,-2.299)	<0.001	5	-0.386 (-0.518,-0.254)	<0.001	4	-2.879 (-3.007,-2.750)	<0.001
**HMSDI regions**									
Male	4	-1.781 (-2.131,-1.430)	<0.001	5	-0.223 (-0.281,-0.164)	<0.001	4	-1.920 (-2.232,-1.607)	<0.001
Female	4	-2.238 (-2.536,-1.940)	<0.001	4	-0.360 (-0.376,-0.345)	<0.001	4	-2.552 (-2.809,-2.294)	<0.001
Both	4	-2.004 (-2.313,-1.694)	<0.001	5	-0.329 (-0.354,-0.304)	<0.001	4	-2.215 (-2.477,-1.952)	<0.001
**MSDI regions**									
Male	4	-1.098 (-1.230,-0.966)	<0.001	4	0.259 (0.224,0.294)	<0.001	4	-1.201 (-1.333,-1.068)	<0.001
Female	5	-1.815 (-1.938,-1.692)	<0.001	5	0.050 (0.030,0.071)	<0.001	5	-2.075 (-2.209,-1.940)	<0.001
Both	5	-1.433 (-1.551,-1.315)	<0.001	5	0.143 (0.094,0.192)	<0.001	5	-1.598 (-1.720,-1.475)	<0.001
**LMSDI regions**									
Male	4	-0.846 (-1.093,-0.598)	<0.001	5	0.151 (0.122,0.180)	<0.001	4	-0.903 (-1.163,-0.642)	<0.001
Female	2	-1.098 (-1.187,-1.009)	<0.001	5	-0.013 (-0.041,0.015)	0.369	2	-1.196 (-1.292,-1.099)	<0.001
Both	2	-0.993 (-1.101,-0.885)	<0.001	5	0.073 (0.040,0.106)	<0.001	2	-1.070 (-1.184,-0.956)	<0.001
**LSDI regions**									
Male	1	-1.062 (-1.100,-1.023)	<0.001	4	-0.291 (-0.307,-0.276)	<0.001	1	-1.100 (-1.139,-1.061)	<0.001
Female	3	-0.903 (-0.966,-0.84)	<0.001	5	-0.313 (-0.354,-0.272)	<0.001	3	-0.953 (-1.025,-0.881)	<0.001
Both	2	-0.992 (-1.028,-0.955)	<0.001	5	-0.295 (-0.316,-0.273)	<0.001	2	-1.040 (-1.079,-1.001)	<0.001
**China**									
Male	4	-1.459 (-1.696,-1.221)	<0.001	5	0.610 (0.575-0.646)	<0.001	4	-1.601 (-1.841,-1.361)	<0.001
Female	5	-2.333 (-2.580,-2.085)	<0.001	5	0.460 (0.432-0.488)	<0.001	5	-2.741 (-2.991,-2.49)	<0.001
Both	5	-1.832 (-2.062,-1.602)	<0.001	5	0.521 (0.438-0.605)	<0.001	5	-2.082 (-2.328,-1.836)	<0.001

Js – Joinpoints, CI – confidence interval, AAPC – average annual percent change, DALY – disability-adjusted life yeas, YLD – lives with disability, YLL – years of life lost, SDI – socio-demographic index, HSDI – high SDI, HMSDI – high-middle SDI, MSDI – middle SDI, LMSDI – low-middle SDI, LSDI – low SDI

**Table 6:** AAPC differences of DALY, YLD, YLL of stroke in China and the different SDI regions over the world from 1990 to 2019

		DALY		YLD		YLL	
**Location**		**Differences (95% CI)**	***P*-value**	**Differences (95% CI)**	***P*-value**	**Differences (95% CI)**	***P*-value**
China	Global	-0.354 (-0.626,-0.081)	0.011	0.682 (0.595-0.770)	<0.001	-0.449 (-0.732,-0.166)	0.002
China	HSDI	0.552 (0.307-0.797)	<0.001	0.907 (0.751-1.064)	<0.001	0.797 (0.519-1.074)	<0.001
China	HMSDI	0.172 (-0.214,0.557)	0.383	0.850 (0.736-0.938)	<0.001	0.133 (-0.227,0.493)	0.470
China	MSDI	-0.399 (-0.658,-0.141)	0.002	0.378 (0.281-0.476)	<0.001	-0.484 (-0.759,-0.209)	0.001
China	LMSDI	-0.840 (-1.094,-0.586)	<0.001	0.449 (0.358-0.539)	<0.001	-1.013 (-1.284,-0.741)	<0.001
China	LSDI	-0.841 (-1.073,-0.608)	<0.001	0.816 (0.730-0.903)	<0.001	-1.042 (-1.291,-0.793)	<0.001
Global	HSDI	0.905 (0.736-1.075)	<0.001	0.225 (0.090-0.360)	0.001	1.246 (1.056-1.436)	<0.001
Global	HMSDI	0.525 (0.183-0.867)	0.003	0.168 (0.132-0.204)	<0.001	0.582 (0.284-0.880)	<0.001
Global	MSDI	-0.046 (-0.234,0.142)	0.633	-0.304 (-0.360,-0.248)	<0.001	-0.035 (-0.221,0.151)	0.711
Global	LMSDI	-0.486 (-0.668,-0.304)	<0.001	-0.234 (-0.276,-0.192)	<0.001	-0.563 (-0.744,-0.383)	<0.001
Global	LSDI	-0.487 (-0.638,-0.336)	<0.001	0.134 (0.100-0.168)	<0.001	-0.593 (-0.738,-0.448)	<0.001
HSDI	HMSDI	0.380 (0.059-0.701)	0.020	0.057 (-0.077,0.192)	0.406	0.664 (0.371-0.956)	<0.001
HSDI	MSDI	-0.951 (-1.097,-0.806)	<0.001	-0.529 (-0.670,-0.388)	<0.001	-1.281 (-1.458,-1.103)	<0.001
HSDI	LMSDI	-1.391 (-1.529,-1.254)	<0.001	-0.459 (-0.595,-0.322)	<0.001	-1.809 (-1.981,-1.637)	<0.001
HSDI	LSDI	-1.392 (-1.485,-1.300)	<0.001	-0.091 (-0.225,0.043)	0.182	-1.839 (-1.973,-1.705)	<0.001
HMSDI	MSDI	-0.571 (-0.902,-0.240)	0.001	-0.472 (-0.527,-0.417)	<0.001	-0.617 (-0.907,-0.327)	<0.001
HMSDI	LMSDI	-1.011 (-1.339,-0.684)	<0.001	-0.402 (-0.443,-0.360)	<0.001	-1.145 (-1.432,-0.859)	<0.001
HMSDI	LSDI	-1.012 (-1.324,-0.701)	<0.001	-0.034 (-0.067,-0.001)	0.042	-1.175 (-1.441,-0.909)	<0.001
MSDI	LMSDI	0.440 (0.280-0.600)	<0.001	-0.070 (-0.130,-0.011)	0.021	0.528 (0.361-0.696)	<0.001
MSDI	LSDI	0.441 (0.318-0.565)	<0.001	-0.438 (-0.491,-0.384)	<0.001	0.558 (0.430-0.687)	<0.001
LMSDI	LSDI	-0.001 (-0.115,0.113)	0.984	0.368 (0.328-0.407)	<0.001	-0.030 (-0.150,0.091)	0.627

CI – confidence interval, AAPC – average annual percent change, DALY – disability-adjusted life yeas, YLD – lives with disability, YLL – years of life lost, SDI – socio-demographic index, HSDI – high SDI, HMSDI – high-middle SDI, MSDI – middle SDI, LMSDI – low-middle SDI, LSDI – low SDI

#### YLD

The final selected JM for the age-standardised YLD rate of stroke indicated that all regions exhibited five joinpoints (**Table 5**). The HSDI regions showed the largest decline in the YLD rate of stroke from 1990 to 2019 (AAPC = -0.386, 95% CI = -0.518 to -0.254, *P* < 0.001), whereas China experienced the largest increase from 1990 to 2019 (AAPC = 0.521, 95% CI = 0.438 to 0.605, *P* < 0.001; **Table 5** and **Figure 1**, panel E). The results of the pairwise comparison analysis showed that the difference in the AAPC for the age-standardised YLD rate of stroke from 1990 to 2019 was not statistically significant between the HSDI and HMSDI/LSDI regions (**Table 6** and **Figure 2**, panel E).

#### YLL

The final selected JM for the age-standardised YLL rate of stroke revealed five joinpoints in the global, China, and MSDI regions, four joinpoints in the HSDI and HMSDI regions, and two joinpoints in the LMSDI and LSDI regions (**Table 5**). The HSDI regions experienced the greatest reduction in the YLL rate of stroke from 1990 to 2019 (AAPC = -2.879, 95% CI = -3.007 to -2.750, *P* < 0.001), with a greater drop from 2004 to 2007 in China than in the other regions (**Table 5** and **Figure 1**, panel F). The results of the pairwise comparison analysis indicated that the difference in AAPC for the age-standardised YLL rate of stroke from 1990 to 2019 was not statistically significant between the China and HMSDI regions, the global and MSDI regions, or between LMSDI and LSDI regions (**Table 6** and **Figure 2**, panel F).

### Analysis on the relationship between stroke burden and SDI

**Figure 3:**
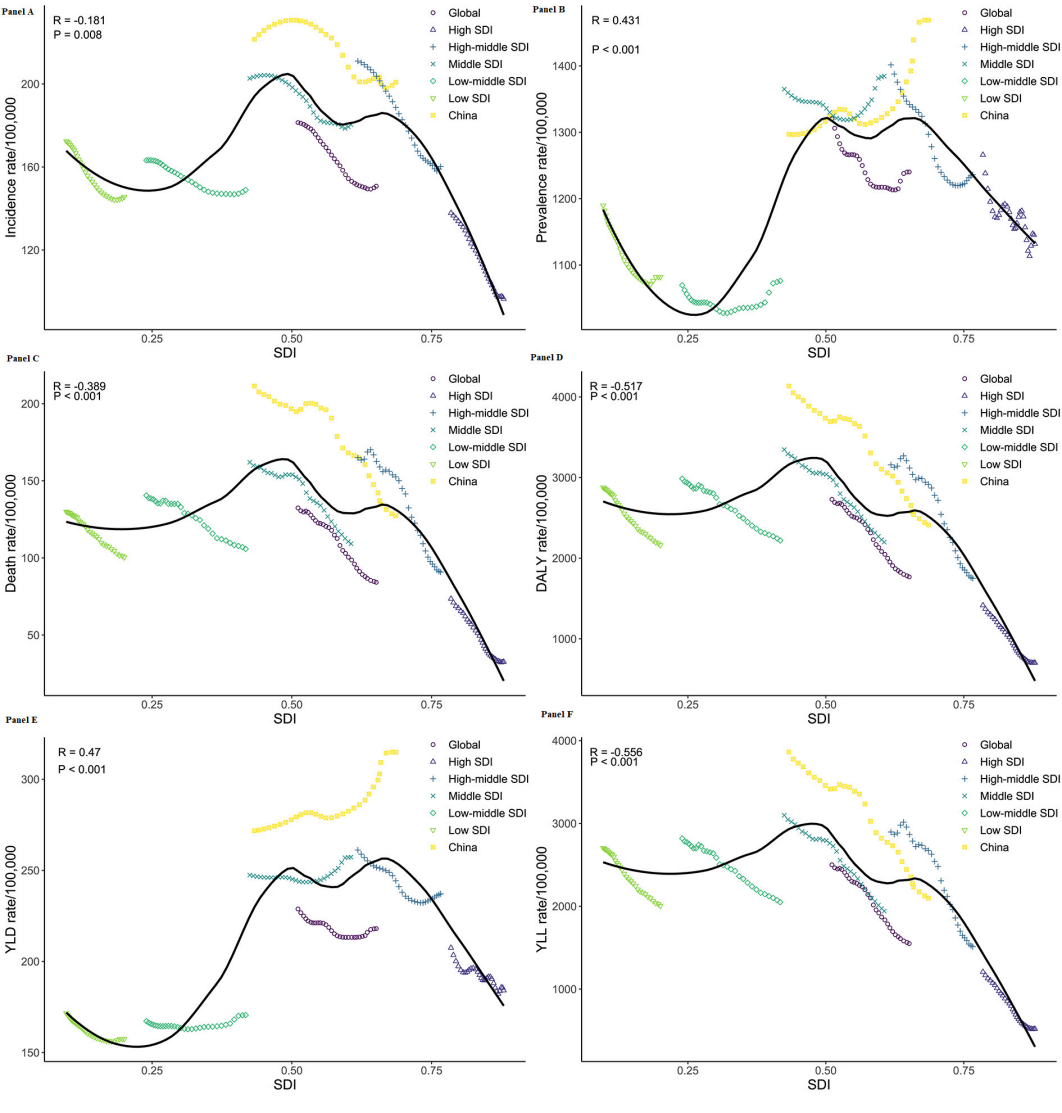
Relationship between burden of stroke and socio-demographic index in different SDI regions over the world from 1990 to 2019. **Panel A.** Relationship between ASIR of stroke and socio-demographic index over the world. **Panel B.** Relationship between ASPR of stroke and socio-demographic index over the world. **Panel C.** Relationship between ASDR of stroke and socio-demographic index over the world. **Panel D.** Relationship between the age-standardised DALY rate of stroke and socio-demographic index over the world. **Panel E.** Relationship between the age-standardised YLD rate of stroke and socio-demographic index over the world. **Panel F.** Relationship between the age-standardised YLL rate of stroke and socio-demographic index over the world.

**Figure 4:**
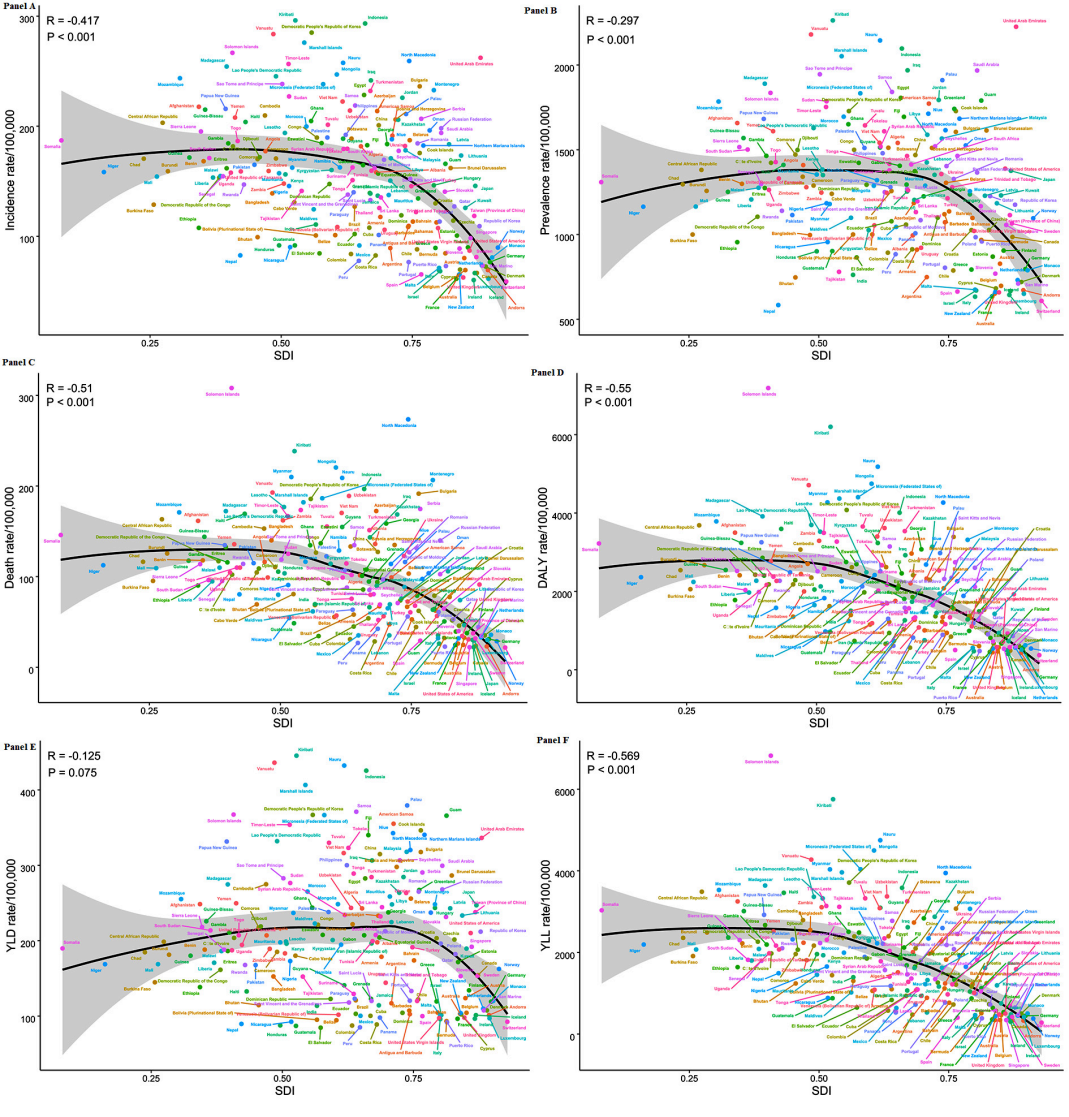
Relationship between burden of stroke and SDI for 204 countries and territories in 2019. **Panel A.** Relationship between ASIR of stroke and SDI for 204 countries and territories. **Panel B.** Relationship between ASPR of stroke and SDI for 204 countries and territories. **Panel C.** Relationship between ASDR of stroke and SDI for 204 countries and territories. **Panel D.** Relationship between the age-standardised DALY rate of stroke and SDI for 204 countries and territories. **Panel E.** Relationship between the age-standardised YLD rate of stroke and SDI for 204 countries and territories. **Panel F.** Relationship between the age-standardised YLL rate of stroke and SDI for 204 countries and territories.

**Figure 3** and **Figure 4** showed the stroke burden of different SDI regions and nationals in relation to SDI vs. the expected level for each location based on SDI. The ASIR and age-standardised YLD rates in the LSDI, MSDI, and HSDI regions were highly consistent with the expected trends (**Figure 3**, panels A and E). The ASPR in the LSDI and HSDI regions were highly consistent with the expected trends (**Figure 3**, panel B). The ASDR and age-standardised DALY/YLL rates in the HSDI regions were highly consistent with the expected trends (**Figures 3**, panels C, D and F). The observed results in other regions varied widely from those expected, with the global values staying significantly below expectations and those in China staying well above expectations.

There was a correlation between all age-standardised rates of stroke in different regions and SDI, with the ASIR, ASDR, and age-standardised DALY/YLL rates inversely correlated with SDI (**Figure 3**, panel A, C, D and F), and the ASPR and age-standardised YLD rates positively correlated with SDI (**Figure 3**, panels B and E). In 2019, all age-standardised rates for stroke at the national level were inversely correlated with SDI, but there were some exceptions (ASIR: R = -0.417, *P* < 0.001; ASPR: R = -0.297, *P* < 0.001; ASDR: R = -0.510, *P* < 0.001; DALY: R = -0.550, *P* < 0.001; YLD: R = -0.125, *P* = 0.075; YLL: R = -0.569, *P* < 0.001; **Figure 4****,** panels A, B, C, D, E and F).

## DISCUSSION

Globally, the overall change in various indicators of stroke burden from 1990 to 2019 showed a downward trend; however, the magnitude of change varied across indicators. Notably, the downward trend in females was more pronounced than that in males. While we acknowledge the important role of global health advocacy as exemplified by events such as “World Stroke Day” it is worth noting that the observed downward trend in females may also reflect differences in self-discipline and health behaviours [27,28]. Other significant factors contributing to the observed trends in stroke burden include socioeconomic and environmental factors and access to health care.

The results observed in the HSDI, HMSDI, and LSDI regions were similar to the global findings, with the HSDI and HMSDI regions consistently exhibiting the most significant downward trend regardless of the stroke burden index used, ranking among the top two. Although the trend of stroke burden in LSDI regions has shown a decrease, with a more pronounced decline among males than among females, the rate of decline is significantly slower than that in HSDI regions. Variations in health care infrastructure, early identification of stroke-related risk factors, and effective control measures may contribute to the observed differences in trends between regions with different levels of socioeconomic development [29,30]. Most countries in the HSDI and HMSDI regions have established comprehensive surveillance systems for cardiovascular and cerebrovascular disease risk factors, enabling better control of risk factors associated with stroke [13]. Interestingly, the LSDI regions exhibited a decrease in ASIR, which was second only to the global trend, and the downward trend was more pronounced compared with the MSDI and LMSDI regions. This may be related to the increasing attention paid by numerous countries in the LSDI region to primary and secondary stroke prevention, while the influence of environment and culture cannot be ruled out [31]. Certainly, we must acknowledge that the level of economic development in a country may influence disease surveillance. For example, it is possible that more developed countries with more funding and better health care can detect more subtle cases of stroke and attribute them to national statistics, whereas those with less developed health care can only attribute very severe cases and case fatalities, significantly reducing the reported incidence rates in these regions.

Notably, in the LMSDI and MSDI regions and in China, both the ASPR and age-standardised YLD rates show an increasing trend. However, in these regions, the age-standardised incidence and YLD rates of stroke among females have exhibited a decreasing trend. The main reasons for this trend may include population growth and aging, as well as a significant increase in several critical risk factors such as ambient particulate pollution, high systolic blood pressure, high fasting blood glucose, and alcohol consumption [32]. Although China falls within the MSDI region, its ASIR, ASPR, and age-standardised YLD rates are much higher than the regional averages in that area. This could be attributed to the impact of improved stroke survival rates [33] as well as China's large population base and insufficient coverage of stroke-prevention measures [34]. It is essential to note that the decline in ASDR, age-standardised DALY, and YLL rates of stroke in China were second only to those in the HMSDI region. This can be attributed to the continuous improvement in China's medical capabilities, ongoing enhancement of the health care infrastructure, and increasing expertise of health care professionals [35]. The descending order of ASDR, age-standardised DALY, and YLL rates in stroke across all regions was as follows: HSDI, HMSDI, China, Global, MSDI, LMSDI, and LSDI. This fully reflects the impact of SDI on the ASDR and age-standardised DALY and YLL rates for stroke [36]. Therefore, it is important to acknowledge the impact of social development on health disparities across countries [37]. Differences in social development can contribute to variations in stroke burden, emphasising the need for targeted interventions and policies to address these disparities.

The results of pairwise comparisons in different regions showed that the AAPC difference in stroke burden in most regions was statistically significant. This may provide strong evidence that the degree of social development affects stroke burden. From the overall trends from 1990 to 2019, this study found that the ASIR, ASDR, and age-standardised DALY and YLL rates in patients with stroke were inversely correlated with the SDI. The higher the SDI, the lower the ASIR, ASDR, age-standardised DALY, and YLL rates in patients with stroke. This may be due to the greater focus on education, quality of life, and individual health awareness in these regions. Moreover, the roles of social and economic development in driving advancements in medical standards should not be overlooked [38]. The ASPR and age-standardised YLD rates were positively correlated with the SDI. The increase in ASPR and age-standardised YLD rates is not solely attributed to population growth and aging, but is also linked to a heightened focus on stroke rehabilitation in regions with higher SDIs, driven by improvements in treatment levels [39]. We also observed an inverse correlation between stroke burden at the national level and the SDI in 2019, although there were some exceptions. This finding further strengthens the relationship between SDI and stroke burden and emphasises the impact of SDI on stroke burden.

Therefore, it is crucial to emphasise the need for tailored public health interventions based on different levels of SDI to ensure improved health care services for patients with stroke [40]. Public health policies and decision makers should prioritise the design and implementation of effective interim policies, considering varying SDI levels, and formulate concrete and actionable measures to form control strategies [41]. Education is of the utmost importance, and countries should strengthen their educational efforts in accordance with their SDI levels to enhance health awareness [42]. As countries pursue social and economic development, they should address the burden of stroke and implement relevant prevention and control policies. For instance, initiatives like “Health of China 2030” [43] and “China’s Medium and Long-Term Plan for the Prevention and Treatment of Chronic Diseases (2017-2025)” [44] have been introduced by the Chinese government. However, the stroke burden in China remains a significant contributor to the global stroke burden. Therefore, there is a need to not only strengthen the control of high-risk factors but also elucidate the underlying mechanisms and allocate available resources in a targeted manner [13]. Stroke management should be performed in all directions in a complex system to effectively reduce the burden of stroke [45]. This is a long and challenging task that is essential for addressing stroke.

Based on the GBD 2019 and SDI data, this study comprehensively analysed and discussed the changing trends and differences in stroke burden between China and different SDI regions worldwide from 1990 to 2019. These findings may provide a useful reference for future policies that consider targeted stroke-prevention strategies. However, this study has some limitations. First, the data used were based on sample information and are not necessarily representative of the entire country or region studied. Second, the quality and validity of the GBD data cannot be guaranteed in some underdeveloped regions. Moreover, because the data set for this study was obtained prior to the Covid-19 pandemic in 2019, the potential impact of the Covid-19 pandemic on stroke burden could not be analysed. In future research, we will include factors such as the level of economic development in each country as covariates to further explore the trends in the burden of stroke across countries and explain the remaining variations. In addition, future studies should include data from the pandemic period and compare the pre-pandemic and pandemic periods to identify significant variations in the stroke burden.

## CONCLUSIONS

In summary, there were significant differences in the stroke burden across various regions with varying SDI levels from 1990 to 2019. The ASPR and attributable disability burden of stroke remain substantial in different SDI regions, making stroke a major contributor to the overall disease burden. The burden of stroke originates primarily in the high-middle, middle, and low SDI regions. Despite some exceptions, the burden of stroke is inversely correlated with the SDI at the national level. The severe burden of stroke highlights the importance of primary and secondary stroke-prevention strategies. Therefore, future strategies to prevent and reduce the burden of stroke should be formulated and implemented according to the SDI of each country. Our findings provide a useful reference for targeted stroke-prevention strategies at different regional and national SDI levels.
